# Development and validation of machine learning models for predicting prognosis and guiding individualized postoperative chemotherapy: A real-world study of distal cholangiocarcinoma

**DOI:** 10.3389/fonc.2023.1106029

**Published:** 2023-03-15

**Authors:** Di Wang, Bing Pan, Jin-Can Huang, Qing Chen, Song-Ping Cui, Ren Lang, Shao-Cheng Lyu

**Affiliations:** Department of Hepatobiliary Surgery, Beijing Chao-Yang Hospital Capital Medical University, Beijing, China

**Keywords:** distal cholangiocarcinoma, DeepSurv, AFR, machine learning, risk stratification, individualized treatment, post-operative chemotherapy

## Abstract

**Background:**

Distal cholangiocarcinoma (dCCA), originating from the common bile duct, is greatly associated with a dismal prognosis. A series of different studies based on cancer classification have been developed, aimed to optimize therapy and predict and improve prognosis. In this study, we explored and compared several novel machine learning models that might lead to an improvement in prediction accuracy and treatment options for patients with dCCA.

**Methods:**

In this study, 169 patients with dCCA were recruited and randomly divided into the training cohort (n = 118) and the validation cohort (n = 51), and their medical records were reviewed, including survival outcomes, laboratory values, treatment strategies, pathological results, and demographic information. Variables identified as independently associated with the primary outcome by least absolute shrinkage and selection operator (LASSO) regression, the random survival forest (RSF) algorithm, and univariate and multivariate Cox regression analyses were introduced to establish the following different machine learning models and canonical regression model: support vector machine (SVM), SurvivalTree, Coxboost, RSF, DeepSurv, and Cox proportional hazards (CoxPH). We measured and compared the performance of models using the receiver operating characteristic (ROC) curve, integrated Brier score (IBS), and concordance index (C-index) following cross-validation. The machine learning model with the best performance was screened out and compared with the TNM Classification using ROC, IBS, and C-index. Finally, patients were stratified based on the model with the best performance to assess whether they benefited from postoperative chemotherapy through the log-rank test.

**Results:**

Among medical features, five variables, including tumor differentiation, T-stage, lymph node metastasis (LNM), albumin-to-fibrinogen ratio (AFR), and carbohydrate antigen 19-9 (CA19-9), were used to develop machine learning models. In the training cohort and the validation cohort, C-index achieved 0.763 *vs.* 0.686 (SVM), 0.749 *vs.* 0.692 (SurvivalTree), 0.747 *vs.* 0.690 (Coxboost), 0.745 *vs.* 0.690 (RSF), 0.746 *vs.* 0.711 (DeepSurv), and 0.724 *vs.* 0.701 (CoxPH), respectively. The DeepSurv model (0.823 *vs.* 0.754) had the highest mean area under the ROC curve (AUC) than other models, including SVM (0.819 *vs.* 0.736), SurvivalTree (0.814 *vs.* 0.737), Coxboost (0.816 *vs.* 0.734), RSF (0.813 *vs.* 0.730), and CoxPH (0.788 *vs.* 0.753). The IBS of the DeepSurv model (0.132 *vs.* 0.147) was lower than that of SurvivalTree (0.135 *vs.* 0.236), Coxboost (0.141 *vs.* 0.207), RSF (0.140 *vs.* 0.225), and CoxPH (0.145 *vs.* 0.196). Results of the calibration chart and decision curve analysis (DCA) also demonstrated that DeepSurv had a satisfactory predictive performance. In addition, the performance of the DeepSurv model was better than that of the TNM Classification in C-index, mean AUC, and IBS (0.746 *vs.* 0.598, 0.823 *vs.* 0.613, and 0.132 *vs.* 0.186, respectively) in the training cohort. Patients were stratified and divided into high- and low-risk groups based on the DeepSurv model. In the training cohort, patients in the high-risk group would not benefit from postoperative chemotherapy (p = 0.519). In the low-risk group, patients receiving postoperative chemotherapy might have a better prognosis (p = 0.035).

**Conclusions:**

In this study, the DeepSurv model was good at predicting prognosis and risk stratification to guide treatment options. AFR level might be a potential prognostic factor for dCCA. For the low-risk group in the DeepSurv model, patients might benefit from postoperative chemotherapy.

## Introduction

Cholangiocarcinoma (CCA) includes intrahepatic CCA, perihilar CCA, and distal CCA (dCCA). Among them, dCCA, originating from the common biliary duct, is an aggressive tumor that probably accounts for 20%%–30% of all CCA cases (1, 2). Most patients with dCCA usually have advanced disease at presentation due to difficulties in early diagnosis (3). Surgical resection remains the primary treatment strategy for dCCA, such as pancreaticoduodenectomy (PD) with standard lymphadenectomy. The survival outcome of dCCA is still dismal because of the relatively low resection rate and the high relapse rate after the operation, and the 5-year overall survival rate is approximately between 20% and 50% (4, 5). Although reports have suggested that surgical resection combined with postoperative chemotherapy benefits patients with dCCA, an ideal medical model, which could lead to improvement in prediction accuracy and treatment options, remains of paramount importance (6).

The Union for International Cancer Control (UICC) TNM Classification is a globally recognized standard for classifying the extent of the spread of cancer, which records the primary and regional nodal extent of the tumor and the absence or presence of metastases. Nevertheless, the TNM Classification could not accurately predict patients’ prognosis once they received multimodality treatment. The TNM Classification only included anatomical prognostic factors and cannot incorporate non-anatomical factors associated with prognosis.

Machine learning is the name given to both the academic discipline and the collection of techniques that allow computers to undertake complex tasks. As an academic discipline, machine learning comprises elements of mathematics, statistics, and computer science. The application of machine learning mainly benefits diagnosis and outcome prediction in the medical field (7). Machine learning algorithms have been successfully applied to classify skin cancer by dermatologists (8) and to predict the progression from pre-diabetes to type II diabetes (9). Several machine learning models have been reported, including random survival forest (RSF) (10), support vector machine (SVM) (11), and DeepSurv (12), although inconsistency remains, and model-building approaches, effect estimates, and the overall accuracy and validation of these prediction models vary to the point that a consensus has not been reached.

In this study, we constructed several different machine learning models and canonical logistic Cox proportional hazards (CoxPH) and evaluated their predictive performance. The aim was to demonstrate the effectiveness of machine learning and guide individualized treatment options for dCCA patients.

## Materials and methods

### Patients and dataset

The clinical data of patients were collected through a retrospective review of medical records. Eligible patients were those who underwent PD for dCCA between October 2011 and December 2021. Patient data were retrieved from the hospital database. Patients were randomly divided into the training cohort and the validation cohort. The study was performed following the tenets of the Declaration of Helsinki (as revised in 2013) and was approved by the Ethics Committee of Beijing Chao-Yang Hospital Capital Medical University (No. 2020-D-301).

### Data collection

We collected the demographic information of dCCA patients, such as age, gender, and history of smoking or diabetes. The preoperative blood values included white blood cell (WBC), hemoglobin (Hb), platelet (PLT), albumin (Alb), aspartate transaminase (AST), alanine transaminase (ALT), total bilirubin (TBIL), γ-glutamyl transpeptidase (GGT), plasma fibrinogen (Fib), carbohydrate antigen 19-9 (CA19-9), and carcinoembryonic antigen (CEA). Albumin-to-fibrinogen ratio (AFR) was calculated by dividing the Alb concentration by the Fib concentration. Peri-operative data included tumor differentiation, lymph node metastasis (LNM), T-stage, intraoperative blood loss, and operative duration. Postoperative chemotherapy and survival outcome were also recorded.

### Flow chart of study

In this study, patients with dCCA were recruited and randomly divided into the training cohort and the validation cohort. Variables identified as independently associated with the primary outcome by least absolute shrinkage and selection operator (LASSO) regression, the RSF algorithm, and univariate and multivariate Cox regression analyses were introduced to establish the following different machine learning models and canonical regression model: SVM, SurvivalTree, Coxboost, RSF, DeepSurv, and CoxPH. The performance of models was measured and compared with mean AUC, IBS, and C-index following cross-validation. The machine learning model with the best performance was screened out and compared with the TNM Classification by mean AUC, IBS, and C-index. The DeepSurv model was further evaluated by calibration chart and decision curve analysis (DCA). Finally, patients were stratified based on the model with the best performance to assess whether they benefited from postoperative chemotherapy through the log-rank test (**Supplementary Figure 1**).

### Modeling process

*SVM* is a supervised machine learning algorithm that is used for classification or regression. The objective of SVM is to find the hyperplane in high-dimensional space that best separates the data into classes. This hyperplane is called the maximum margin hyperplane and is selected based on the idea of maximizing the margin, which is the distance between the hyperplane and the closest training samples, called support vectors (13). The goal of SVM is to find the hyperplane that maximizes this margin while correctly separating the classes. Once the hyperplane is determined, new data can be classified by finding which side of the hyperplane it falls on. The formula is as follows:


$$b^{*}=y_{j}-\sum_{i=1}^{1}\alpha_{i}^{*}y_{i}\left(x_{i}x_{j}\right)$$

*SurvivalTree* is a type of machine learning algorithm that is used to model and predict time-to-event data, also known as survival analysis. The main objective of SurvivalTree is to estimate the survival function, which represents the probability that an individual will survive past a given time point, given their specific characteristics or features (14). SurvivalTree is a tree-based algorithm, meaning it builds a tree-like structure to represent the relationships between different features and survival time. The algorithm splits the sample into different subgroups based on their features and predicts the survival function for each subgroup. By doing this, the algorithm can capture complex non-linear relationships between features and survival time and make accurate predictions for new individuals. The formula can be expressed as:


S(t)=e^(−h(t)*γ(t))

*Coxboost* is a machine learning algorithm that combines the ideas of boosting and the Cox proportional hazards model, which is a popular method in survival analysis. The main objective of Coxboost is to model and predict time-to-event data, also known as survival analysis. Coxboost uses a combination of boosting and the Cox proportional hazards model to make predictions. Boosting is an ensemble learning technique that combines multiple weak learners to form a strong prediction model. In Coxboost, boosting is used to improve the performance of the Cox proportional hazards model by combining multiple models into a single model that has improved accuracy (15). The formula can be expressed as:


$$h\left(t\right)=h_{0}\left(t\right)*\text{exp}\left(\sum^{}\theta i*f\left(t,xi\right)\right)$$

*RSF* is a machine learning algorithm that is used to model and predict time-to-event data, also known as survival analysis. The main objective of RSF is to estimate the survival function, which represents the probability that an individual will survive past a given time point, given their specific characteristics or features. RSF is an ensemble learning algorithm that combines multiple decision trees to form a random forest. Decision trees are tree-based algorithms that split the sample into different subgroups based on their features and predict the survival time for each subgroup. By combining multiple decision trees, RSF can capture complex non-linear relationships between features and survival time and make accurate predictions for new individuals (16). The test statistic function for the Z-value to accept the null hypothesis is as follows:


$$Z=\frac{\sum_{i=1}^{k}\left(O_{1,\;i}-E_{1,i}\right)}{\sqrt{\sum_{i=1}^{k}V_{i}}}-N\left(0,1\right)$$

*CoxPH* is a statistical method that is commonly used in survival analysis to model the relationship between covariates and time-to-event data. The main objective of CoxPH is to estimate the hazard function, which represents the instantaneous risk of an event occurring at a given time. CoxPH assumes that the hazard function is proportional for different individuals, meaning that the hazard ratio between two individuals is constant over time. This allows for the estimation of the hazard function using a simple linear regression model, where the hazard ratio between two individuals can be estimated as the exponential of the difference in their covariate values. The model can be written as follows (17):


$$Inh\left(t\right)=In\;h_{0}\left(t\right)+b_{1}x_{1}+\ldots +b_{p}x_{p}$$

*The DeepSurv model* was established based on the proposal by Katzman et al. to predict the prognosis of dCCA. DeepSurv is a multilayer neural network, including input, hidden, and output layers. This model imitates the actual clinical patients’ risk value and has tremendous generalization performance. DeepSurv contains weight decay regularization, batch normalization, and dropout, which can prevent overfitting to some extent. The loss function in the DeepSurv model is defined as Cox partial likelihood with constraints, the formula of which is as follows:


$$l\left(\theta \right)=-\frac{1}{N_{E}}\sum_{i,E_{i}=1}\left(\hat{h_{\theta }}\left(x\right)-log\sum_{j\in R\left(T_{i}\right)}e^{\hat{h_{\theta }}\left(x\right)}\right)+\mathrm{\alpha ||}\mathrm{\theta ||}\begin{array}{c} 2\\ 2 \end{array}$$

We think that the smaller the value of loss function in this model, the better the stability. The Adam optimization algorithm was used and obtained current optimal parameters (18). **Supplementary Table 1** shows the hyperparameters of DeepSurv. More detailed information about DeepSurv can be found on the website (https://github.com/jaredleekatzman/DeepSurv) (19).

### Statistical analysis

Continuous variables were shown as mean ± standard deviation (SD) or median (interquartile range [IQR]). Categorical variables are presented as a percentage. Statistical methods are the t-test, Mann–Whitney U-test, and chi-squared test. Kaplan–Meier analysis and log-rank testing were operated using the lifelines module by Python. AFR was evaluated using “R-package Survminer” to obtain the best cutoff value. Eventually, the predictive ability was analyzed and compared between the machine learning model with the best performance and the CoxPH model. The accuracy of prognostic prediction models was assessed using the C-index, calibration chart, DCA, IBS, and AUC in the training and validation cohorts. Statistical analyses were performed using R software (version 4.0.4) and Python software (version 3.7.6). These tests of the proposed approaches were double-sided, and the standard of significance in the results was p < 0.05.

## Results

### The clinical characteristics of patients and selection of variables

We searched electronic medical records and identified 169 patients (105 men and 64 women, mean age 65 years, range 29–84 years) diagnosed with dCCA between October 2011 and December 2021 in this study. Among all patients, 113 (66.9%) had medium–high differentiation cancer, 141/169 (83.4%) were at T3/4 stage during presentation, 77 (45.6%) had LNM, and 41 (24.3%) received postoperative chemotherapy. Patients were randomly divided into the training cohort (n = 118) and the validation cohort (n = 51) by 7:3. There were no major differences in the demographic and clinical characteristics of patients between the two cohorts (**Table 1**). The cumulative incidence curves also had no significant difference in the two cohorts using the log-rank test (p = 0.21) (**Supplementary Figure 2**). Based on “R-package Survminer”, the best cutoff value for AFR was 11.26, and patients were divided into two groups: the high-AFR group (AFR > 11.26, n = 103, 60.9%) and the low-AFR group (AFR ≤ 11.26, n = 66, 39.1%). The univariate and multivariate Cox regression analyses showed that patients with low AFR had a dismal prognosis (HR 2.370 *vs.* HR 1.933, 95% CI 1.470–3.779 *vs.* 95% CI 1.144–3.266, respectively). LASSO analysis and RSF also demonstrated that AFR played an important role in prognosis. The Kaplan–Meier survival curve of overall survival (OS) showed that compared with the high-AFR group, patients with dCCA in the low-AFR group had worse OS (**Supplementary Figure 3**).

**Table 1:** Clinical and pathologic characteristics of 169 patients with dCCA.

Characteristics	Training cohort	Validation cohort	p-value
Number of patients, n (%)	118 (69.8)	51 (30.2)	
**Age (years), n (%)**			0.493
≤65	58 (49.2)	28 (54.9)	
>65	60 (50.8)	23 (45.1)	
**Gender, n (%)**			0.914
Male	73 (61.9)	32 (62.7)	
Female	45 (38.1)	19 (37.3)	
**Diabetes, n (%)**			0.240
No	89 (75.4)	34 (66.7)	
Yes	29 (24.6)	17 (33.3)	
**Smoking status, n (%)**			0.280
No	78 (66.1)	38 (74.5)	
Yes	40 (33.9)	13 (25.5)	
**Differentiation, n (%)**			0.165
Medium–high	75 (63.6)	38 (74.5)	
Poor	43 (36.4)	13 (25.5)	
**LNM, n (%)**			0.352
No	67 (56.8)	25 (49)	
Yes	51 (43.2)	26 (51)	
**Postoperative chemotherapy, n (%)**			0.884
No	89 (75.4)	39 (76.5)	
Yes	29 (24.6)	12 (23.5)	
**AFR, n (%)**			0.178
High	50 (42.4)	16 (31.4)	
Low	68 (57.6)	35 (68.6)	
**T, n (%)**			0.270
T1/2	22 (18.6)	6 (11.8)	
T3/4	96 (81.4)	45 (88.2)	
**CA19-9, n (%)**			
≤37	38 (32.2)	16 (31.4)	0.915
>37	80 (67.8)	35 (68.6)	
**WBC (10^9^/L), mean ± SD**	6.3 ± 2.3	6.7 ± 1.9	0.192
**TBIL (μmol/L), median (IQR)**	110.0 (170.3)	158.3 (164.8)	0.387
**GGT (U/L), median (IQR)**	345.0 (650.0)	381.0 (596.0)	0.792
**Hb (g/L), mean ± SD**	117.2 ± 20.4	114.6 ± 24.0	0.794
**PLT (10^9^/L), mean ± SD**	240.3 ± 75.8	257.3 ± 81.9	0.191
**AST (U/L), median (IQR)**	60.0 (83.3)	65.0 (50.0)	0.105
**CEA (U/L), median (IQR)**	2.0 (1.9)	2.4 (2.8)	0.980
**ALT (U/L), median (IQR)**	69.0 (112.0)	68.0 (129.0)	0.450
**Fib (g/L), median (IQR)**	3.4 (1.5)	3.8 (1.2)	0.298
**Alb (g/L), mean ± SD**	35.3 ± 5.8	34.2 ± 5.8	0.246
**Blood loss (ml), median (IQR)**	500.0 (325.0)	500.0 (200.0)	0.487
**Operative time (h), mean ± SD**	10.1 ± 2.2	10.1 ± 2.2	0.929

dCCA, distal cholangiocarcinoma; LNM, lymph node metastasis; CA19-9, carbohydrate antigen 19-9; TBIL, total bilirubin; GGT, γ-glutamyl transpeptidase; Hb, hemoglobin; PLT, blood platelet; ALT, alanine aminotransferase; Alb, albumin; IQR, interquartile range; AFR, albumin-to-fibrinogen ratio; WBC, white blood cell AST, aspartate transaminase; CEA, carcinoembryonic antigen.

### Selecting the variables and establishing the machine learning models

**Figure 1:**
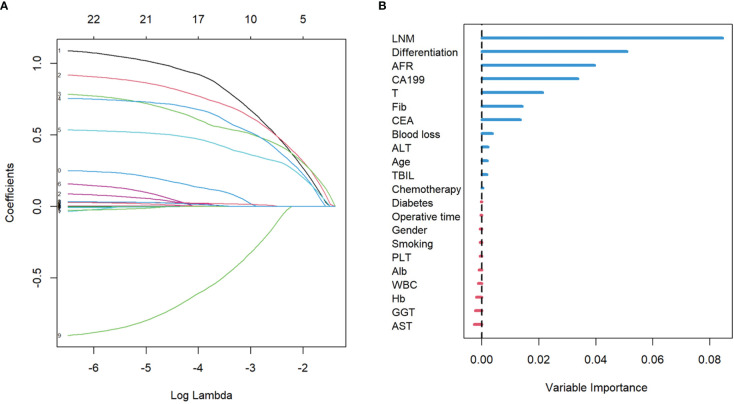
The results of LASSO regression analysis and the RSF plot for models. **(A)** LASSO coefficient profiles of the expression of 22 variables. **(B)** The length of the horizontal axis where each variable is located represents the variable’s contribution to the outcome. LASSO, least absolute shrinkage and selection operator; RSF, random survival forest.

**Figure 2:**
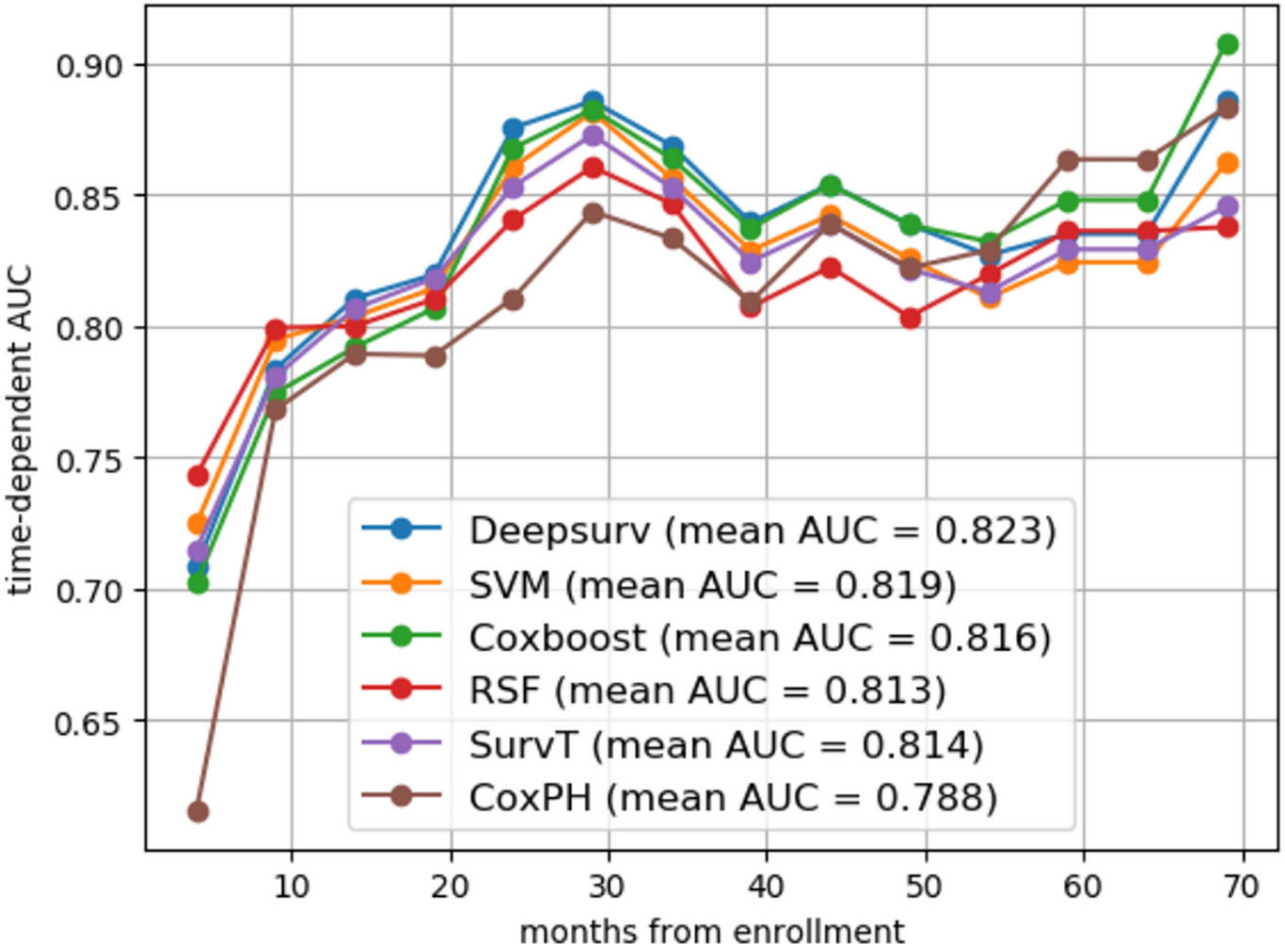
The time-dependent ROC analysis in different models. The time-dependent ROC analysis in SVM, SurvivalTree, Coxboost, RSF, DeepSurv models, CoxPH, and DeepSurv had a higher mean AUC than other models in the training cohort. ROC, receiver operating characteristic; AUC, area under the ROC curve.

From the basic information, laboratory examinations, and peri-operative and postoperative data, 22 characteristics were reduced to five potential predictors. Algorithms, including LASSO regression (**Figure 1A**), RSF (**Figure 1B**), and univariate and multivariate Cox regression analyses (**Tables 2**, **3**), showed that tumor differentiation, T-stage, LNM, AFR, and CA19-9 were prognostic variables identified as independently associated with the primary outcome. Six models, namely, SVM, SurvivalTree, Coxboost, DeepSurv, RSF, and CoxPH, were established based on the identified five prognostic variables. In the training cohort, the predictive performance of the SVM model (0.763) was better than that of the other models, with C-indexes of SurvivalTree (0.749), Coxboost (0.747), DeepSurv (0.746), RSF (0.745), and CoxPH (0.724). However, in the validation cohort, the C-index of DeepSurv (0.711) was better than that of the other models: SVM (0.686), SurvivalTree (0.692), Coxboost (0.690), and RSF (0.690). We also performed time-dependent ROC analysis in different models (**Figure 2**). The results in the training cohort showed that DeepSurv (0.823) had a higher mean AUC than other models, including SVM (0.819), SurvivalTree (0.814), Coxboost (0.816), RSF (0.813), and CoxPH (0.788). In the validation cohort, DeepSurv (0.754) also had a higher mean AUC than the other models, including SVM (0.736), SurvivalTree (0.737), Coxboost (0.734), RSF (0.730), and CoxPH (0.753). Regarding the IBS in the training and validation cohorts, DeepSurv (0.132 *vs.* 0.147) had a lower value than the other models, including SurvivalTree (0.135 *vs.* 0.236), Coxboost (0.141 *vs.* 0.207), RSF (0.140 *vs.* 0.225), and CoxPH (0.145 *vs.* 0.196).

**Table 2:** Univariate Cox regression analyses for predicting OS of patients with dCCA in the training cohort.

Characteristics	Number of patients (%)	HR (95%CI)	p-value
Age (years)			
≤65	58 (49.2)	–	
>65	60 (50.8)	1.611 (1.013–2.564)	0.044^*^
Gender			
Female	45 (38.1)	–	
Male	73 (61.9)	1.042 (0.649–1.673)	0.865
Smoking			
No	78 (66.1)	–	
Yes	40 (33.9)	1.157 (0.717–1.867)	0.549
Diabetes			
No	89 (75.4)	–	
Yes	29 (24.6)	0.919 (0.539–1.567)	0.755
T			
T1/2	22 (18.6)	–	
T3/4	96 (81.4)	3.374 (1.445–7.787)	0.005^**^
LNM			
No	67 (56.8)	–	
Yes	51 (43.2)	2.35 (1.470–3.779)	0.000^***^
AFR			
High	50 (42.4)	–	
Low	68 (57.6)	2.370 (1.445–3.886)	0.001^**^
Differentiation			
High–medium	75 (63.6)	–	
Poor	43 (36.4)	2.195 (1.361–3.541)	0.001^**^
CA19-9 (U/ml)			
≤37	38 (32.2)	–	
>37	80 (67.8)	2.188 (1.293–3.703)	0.004^**^
Chemotherapy			
No	89 (75.4)	–	
Yes	29 (24.6)	0.685 (0.382–1.228)	0.204
**Blood loss (ml)**	118 (100)	1.000 (0.999–1.001)	0.832
**Operative time (h)**	118 (100)	1.067 (0.956–1.191)	0.248
**Fib (mg/dl)**	118 (100)	1.002 (1.000–1.004)	0.052
**Alb (g/L)**	118 (100)	0.996 (0.959–1.035)	0.846
**WBC (10^9^/L)**	118 (100)	1.012 (0.916–1.119)	0.807
**PLT (10^9^/L)**	118 (100)	1.003 (0.999–1.006)	0.103
**Hb (g/L)**	118 (100)	0.998 (0.986–1.010)	0.709
**ALT (U/L)**	118 (100)	1.000 (0.999–1.002)	0.711
**GGT (U/L)**	118 (100)	1.000 (1.000–1.001)	0.812
**AST (U/L)**	118 (100)	1.000 (0.998–1.002)	0.756
**CEA (U/ml)**	118 (100)	1.022 (0.995–1.050)	0.104
**TBIL (μmol/L)**	118 (100)	1.002 (1.000–1.004)	0.112

***p-value <0.001; **p-value <0.01; *p-value <0.05.

LNM, lymph node metastasis; CA19-9, carbohydrate antigen 19-9; TBIL, total bilirubin; GGT, γ-glutamyl transpeptidase; Hb, hemoglobin; PLT, blood platelet; ALT, alanine aminotransferase; AFR, albumin-to-fibrinogen ratio; OS, overall survival; dCCA, distal cholangiocarcinoma; WBC, white blood cell; AST, aspartate transaminase; CEA, carcinoembryonic antigen.

**Table 3:** Multivariate Cox regression analyses for predicting OS of patients with dCCA in the training cohort.

Characteristics	Number of patients (%)	HR (95%CI)	p-value
Age (years)			
≤65	58 (49.2)		
>65	60 (50.8)	0.984 (0.604–1.604)	0.948
T			
T1/2	22 (18.6)		
T3/4	96 (81.4)	2.674 (1.121–6.377)	0.027
LNM			
No	67 (56.8)		
Yes	51 (43.2)	2.074 (1.260–3.414)	0.004^**^
AFR			
High	50 (42.4)		
Low	68 (57.6)	1.933 (1.144–3.266)	0.014^*^
Differentiation			
High–medium	75 (63.6)		
Poor	43 (36.4)	1.840 (1.122–3.017)	0.016^*^
CA19-9 (U/ml)			
≤37	38 (32.2)		
>37	80 (67.8)	1.777 (1.033–3.057)	0.038^*^

**p-value <0.01; *p-value <0.05.

LNM, lymph node metastasis; CA19-9, carbohydrate antigen 19-9; AFR, albumin-to-fibrinogen ratio; OS, overall survival; dCCA, distal cholangiocarcinoma.

**Figure 3:**
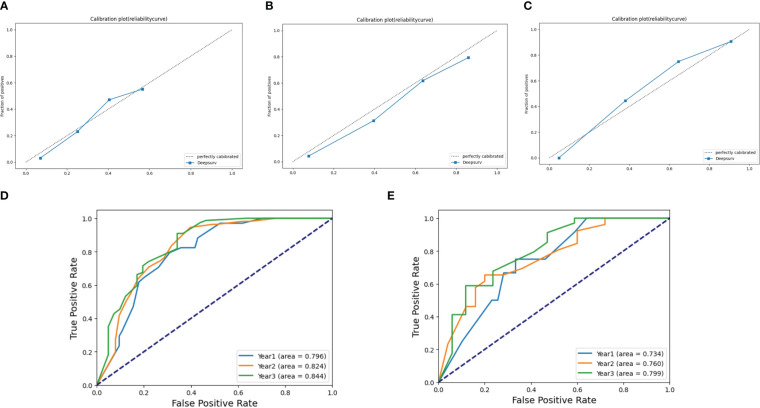
Calibration plots and ROC curve for the DeepSurv model. Calibration plots in **(A)** 1 year, **(B)** 2 years, and **(C)** 3 years in the training cohort. The ROC of 1, 2, and 3 years between the DeepSurv model in the training cohort **(D)** and the validation cohort **(E)**. ROC, receiver operating characteristic.

**Figure 4:**
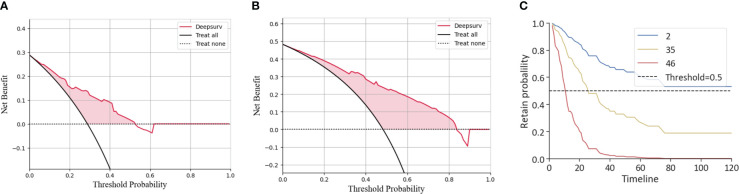
DCA of the DeepSurv model and the individual postoperative prognostic prediction. The 1-year **(B)** and 2-year **(C)** DCA of the DeepSurv model. **(C)** The estimated prognosis of patients in the training cohort. The blue line represents patient 2, the yellow line represents patient 35, and the red line represents patient 46. DCA, decision curve analysis.

The agreement of DeepSurv between predictions and observations in prognosis was assessed using a calibration plot. The 1-, 2-, and 3-year calibration plots showed good agreement between the predictive value and the actual value in the training cohort (**Figures 3A–C**). DeepSurv had good performance in AUC of 1, 2, and 3 years in the training cohort (0.734, 0.824, and 0.844, respectively) (**Figure 3D**) and in the validation cohort (0.734, 0.760, and 0.799, respectively) (**Figure 3E**). DCA was applied to calculate a clinical “net benefit” for the prediction model, and the result of DCA indicated that the DeepSurv model had a better net benefit at most threshold probabilities (**Figures 4A, B**). After comprehensive consideration of the C-index, time-dependent ROC, and IBS, the DeepSurv model was found to have a better predictive performance than the other models, including the traditional Cox model, CoxPH (**Table 4**). We randomly selected three patients for the individual postoperative prognosis demonstration. It showed the individual survival probability of prognosis according to the DeepSurv model (**Figure 4C**).

**Table 4:** Comparison of the bootstrapped C-indexes, mean AUC, and IBS of different models for dCCA patients in the training cohort and the validation cohort.

Characteristics	C-index		Mean AUC		IBS	
	Training cohort	Validation cohort	Training cohort	Validation cohort	Training cohort	Validation cohort
SVM	0.763	0.686	0.819	0.736	–	–
SurvivalTree	0.749	0.692	0.814	0.737	0.135	0.236
RSF	0.745	0.690	0.813	0.730	0.140	0.225
Coxboost	0.747	0.690	0.816	0.734	0.141	0.207
DeepSurv	0.746	0.711	0.823	0.754	0.132	0.147
CoxPH	0.724	0.701	0.788	0.753	0.145	0.196

The DeepSurv model had a more stable prediction ability and a better performance than other models.

AUC, area under the ROC curve; IBS, integrated Brier score; dCCA, distal cholangiocarcinoma.

### Comparison between the DeepSurv model and the TNM classification

As described previously, the TNM Classification is a unified standard and is a prerequisite for ensuring the quality of care, in which oncologists could communicate regarding the cancer extent for individual patients as a basis for decision making on treatment management and individual prognosis, but can also be used to inform and evaluate treatment guidelines, national cancer planning, and research. First, variables involved in the TNM Classification were applied to establish machine learning models. The result of the mean AUC showed that the predictive performance with the time-dependent ROC of DeepSurv (0.613) was better than that of SVM (0.594), SurvivalTree (0.590), Coxboost (0.594), RSF (0.591), and CoxPH (0.594) (**Supplementary Figure 4**). Then, the identified five prognostic variables and TNM Classification variables were employed to develop DeepSurv. Finally, the predictive performance between the DeepSurv model and the TNM Classification was estimated using the value of C-index, mean AUC, and IBS. The results in the training cohort showed that the DeepSurv model is better than the TNM Classification (0.746 *vs.* 0.589, 0.823 *vs.* 0.613, and 0.132 *vs.* 0.186, respectively) (**Supplementary Table 2**). In the validation cohort, the performance of the DeepSurv model was also better than that of the TNM Classification estimated by C-index, mean AUC, and IBS (0.711 *vs.* 0.568, 0.753 *vs.* 0.599, and 0.147 *vs.* 0.172, respectively). The results mentioned above indicated that the DeepSurv model was better at predictive performance and accuracy than the TNM Classification in this study.

### Risk stratification and guidance of individualized chemotherapy for patients with dCCA

**Figure 5:**
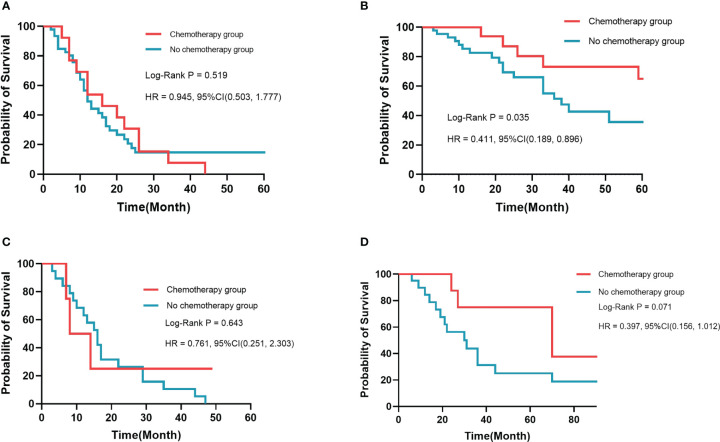
Kaplan–Meier survival analysis in different risk groups. There was no significant difference in prognosis for high-risk patients in the training cohort **(A)** and the validation cohort **(C)**. Patients who received chemotherapy had a better prognosis than those who did not in the training cohort **(B)** and the validation cohort **(D)**.

It is crucial to develop a stratified treatment recommendation to ensure individualized medicine. Patients in this study were stratified into the low-risk group and the high-risk group based on the DeepSurv model in the training cohort and the validation cohort, respectively (**Supplementary Figure 5**). The benefit from chemotherapy was estimated between the high-risk group and the low-risk group by the Kaplan–Meier analysis and log-rank test in the two cohorts. In the training cohort (**Figures 5A, B**), results showed that there was no statistical difference in the prognosis in the high-risk group, regardless of whether receiving chemotherapy or not (p = 0.519). In the low-risk group, patients who received postoperative chemotherapy had a better survival prognosis (p = 0.035). In the validation cohort (**Figures 5C, D**), there was no statistical difference in prognosis in the high-risk group (p = 0.643) and the low-risk group (p = 0.071), regardless of whether receiving chemotherapy or not. However, we still believe that risk stratification based on the DeepSurv model has potential clinical application in which postoperative chemotherapy might benefit patients with dCCA in the low-risk group.

## Discussion

dCCA, originating from the common bile duct and arising from the biliary epithelium, is a heterogeneous and exceptionally aggressive malignant tumor with poor prognosis (20), and the incidence of dCCA is increasing globally. The clinical manifestations of dCCA are frequently non-specific and are related to the biliary obstruction caused by the tumor (21). The silent and asymptomatic nature of dCCA, particularly in its early stages, in combination with its high aggressiveness, intra- and inter-tumor heterogeneity, and chemoresistance, significantly compromises the efficacy of current therapeutic options, contributing to a dismal prognosis (22). Surgery is a potential curative option of reference for early stage tumors. Surgical strategies for dCCA usually require performing a pancreaticoduodenectomy, with the removal of the head of the pancreas, the first part of the duodenum, the gallbladder, and the bile duct (23). However, only 35% of patients are eligible for surgical treatment, and there is a very high rate of postoperative local recurrence (24). The 5-year OS of patients with dCCA is 23% and is slightly higher (27%) if R0 resection is achieved (the median survival after R0 resection is 25 months) (25). Therefore, it is important to increase awareness of this cancer. In the past decade, increasing efforts have been made to understand the complexity of these tumors and to develop new diagnostic tools and therapies that might help to improve patient outcomes (26). Moreover, specific prognostic models must be established for the early diagnosis, prevention, and targeted and personalized treatment options for patients with dCCA (3).

Machine learning is the name given to both the academic discipline and the collection of techniques that allow computers to undertake complex tasks. As an academic discipline, machine learning comprises elements of mathematics, statistics, and computer science. Machine learning techniques are attracting substantial interest from medical researchers and clinicians, which could accommodate different configurations of raw data, assign context weighting, and calculate the predictive power of every combination of variables available to assess diagnostic and prognostic elements. Machine learning algorithms could handle risk profiles that are highly individualized, allowing analysis of disorders with multiple etiologies and incomplete data, as is typical in real clinical settings. Using decision trees, medical researchers could then extract the minimum data necessary to make a diagnosis or therapeutic recommendations. For instance, a feature selection algorithm reduced the number of features (from 29 to 8) necessary for diagnosing autism spectrum disorder (ASD) with 100% accuracy among 612 patients with ASD (27). In this study, the number of patients’ clinical features was also reduced from 21 to 5 after the analysis of the algorithm, which would reduce the time needed to make an accurate diagnosis and improve patient outcomes. We then established algorithms on data from the training cohort and predicted the diagnostic outcome in the validation cohort. We compared the predictive performance between machine learning modes and the traditional Cox model in the training cohort and the validation cohort and found that the DeepSurv model was good at predicting prognosis and risk stratification to guide treatment options.

There is no widely used staging system for dCCA, although it can be staged according to the TNM Classification, which has become the benchmark for classifying patients with cancer, defining prognosis, and determining the best treatment approaches (28, 29). Despite providing a clinically meaningful classification correlated with prognosis (30), the current TNM Classification has some limitations. First, it has limited discriminatory ability between T2 and T3 tumors in surgically resected dCCA (31). T2 tumors include multifocal disease or disease with a vascular invasion that probably reflects disseminated disease, and the OS in patients with these tumors does not differ from the OS in patients with metastatic disease. Second, although size has been included as a prognostic factor for dCCA in the 8th edition of the American Joint Committee on Cancer (AJCC) Cancer Staging Manual, the only cutoff size considered is 5 cm in T1 tumors. Several reports have shown that a 2-cm cutoff value might identify very early tumors with a very low likelihood of dissemination and potentially long-term survival with low recurrence rates (32). Finally, the TNM Classification misses relevant prognostic factors, such as the presence of cancer-related symptoms (abdominal pain or malaise) or the degree of liver function impairment. Notably, Chaiteerakij R et al. proposed a new staging system for dCCA based on tumor size and number, vascular encasement, lymph node and peritoneal metastasis, Eastern Cooperative Oncology Group performance status, and CA19-9 level, which has shown a better performance in predicting survival than the TNM Classification (33). After the analysis in this study, we found that 1) the predictive performance of DeepSurv was better than that of other machine learning models and CoxPH by assessing C-index, mean AUC, and IBS; 2) the identified five prognostic variables by algorithms, including AFR, tumor differentiation, T stage, LNM, and CA19-9, were better than TNM Classification variables at predicting prognosis; and 3) the predictive performance of DeepSurv was better than that of the TNM Classification.

Presently, numerous studies have found that the nutritional status of the tumor patient is one of the key factors influencing the progression of the tumor (34). Malnutrition in cancer patients is a well-recognized phenomenon, especially for patients with digestive system tumors, driven by a combination of reduced food intake, decreased physical activity, and abnormal catabolic–metabolic balance caused by tumors (35). At the same time, inflammation also plays an important role in the pathogenesis of malignant tumors, which is considered to be the seventh characteristic of tumors (36). In daily practice, serum Alb has been used as a simple and reproducible parameter to assess nutrition status, an independent predictor of survival outcome in cancer patients, such as in gastric cancer in which lower serum Alb concentration was associated with worse patient prognosis (37). Xifeng Xu et al. showed that hypoalbuminemia is an independent poor prognostic indicator in patients with non-metastatic breast cancer (38). Inflammation promotes the release of Fib, which further promotes tumor cell proliferation and metastasis by participating in extracellular matrix formation and inducing epithelial–mesenchymal transition (39, 40). Guoying Wang et al. confirmed that elevated levels of Fib predicted poor outcomes in patients with hepatocellular carcinoma (41). Juan Zhao et al. found that high levels of Fib were related to poor prognosis in patients with early stage resectable extrahepatic CCA (42). AFR, which takes both Alb and Fib into account, has been indicated as a prognostic factor for various malignancies, including non-small cell lung cancer (43), chronic lymphocytic leukemia (44), and breast cancer (45). In this study, we also found that AFR was an independent prognostic variable, and the dCCA patients with low AFR might have poor OS.

Even in clinical settings where cancer patients undergo uniform therapeutic regimens, the response and OS rates are highly variable (46). Risk stratification is essential in the evaluation and management of cancer patients. Jianzhen Lin et al. (47) demonstrated that patients with refractory biliary tract carcinomas can derive considerable benefit from receiving personalized therapy guided by molecular profiling. Consequently, it is important to formulate a simple and powerful risk stratification system to identify patients with aggressive cancer courses and assist in optimizing treatment strategies. Machine learning has helped refine risk stratification and triage patients for treatment options. Therefore, we next stratified patients in this study according to the DeepSurv model to validate the prognostic value of this risk stratification model through a retrospective analysis of patients in our hospital that reflected real-world clinical conditions. For low-risk groups, patients with dCCA would benefit from postoperative chemotherapy and have a better survival outcome.

### Study limitation

This study had potential limitations. First, this was a small study with a small sample size in each cohort or group, which could provide results quickly. However, it might also not yield reliable or precise estimates. Another limitation of this small study is that many of the nuances and complexities of machine learning analyses, such as sparsity or high dimensionality, are not well represented in the data. Second, this study represented the data of our single center only, which made it difficult to determine whether a particular outcome was a true finding. Additionally, this was a retrospective study, and an inferior level of evidence, selection bias, and information bias is inevitable. Future studies, preferably with larger patient cohorts from multi-centers and prospective design, should be encouraged to further confirm our preliminary outcomes.

## Conclusions

We constructed accurate prediction models for the survival of patients with dCCA using a novel machine learning platform based on medical data. In this study, the DeepSurv model was good at predicting prognosis and risk stratification to guide treatment options. AFR level might be a potential prognostic factor for dCCA. For the low-risk group in the DeepSurv model, patients might benefit from postoperative chemotherapy. Machine learning has the potential to transform the way that medicine works. We look toward a future of medical research and practice greatly enhanced by the power of machine learning.

## Data availability statement

The original contributions presented in the study are included in the article/**Supplementary Material**. Further inquiries can be directed to the corresponding authors.

## Ethics statement

The authors are responsible for all matters of the work in ensuring that questions related to the accuracy or integrity of any part of the work are appropriately investigated and resolved. The study was conducted in accordance with the Declaration of Helsinki and was reviewed and approved by the Ethics Committee of Beijing Chao-Yang Hospital Capital Medical University (No. 2020-D-301).

## Author contributions

(I) Conception and design: DW and BP. (II) Administrative support: RL and S-CL. (III) Provision of study materials: S-CL and RL. (IV) Collection and assembly of data: J-CH, QC and S-PC. (V) Data analysis and interpretation: DW and BP. (VI) Manuscript writing: DW and BP. (VII) Final approval of manuscript: all authors. All authors made substantial contributions to the conception and design, acquisition of data, or analysis and interpretation of data; took part in drafting the article or revising it critically for important intellectual content; agreed to submit to the current journal; gave final approval of the version to be published; and agreed to be accountable for all aspects of the work.

## Supplementary material

Supplementary Figure 1 Flow chart of this study.

Supplementary Figure 2 Cumulative incidence curves of the training cohort and the validation cohort. Cumulative incidence curve in the training cohort and the validation cohort. There was no statistically significant difference between the survival of the two cohorts using the log-rank test (p = 0.21).

Supplementary Figure 3 The Kaplan–Meier analysis and log-rank test in different AFR groups. The Kaplan–Meier analysis in different AFR groups. There was a statistically significant difference between the survival of the two groups using the log-rank test (p = 0.0013).

Supplementary Figure 4 The time-dependent ROC analysis in TNM Classification. The time-dependent ROC analysis in TNM Classification and the DeepSurv algorithm had the highest mean AUC.

Supplementary Figure 5 The Kaplan–Meier analysis and log-rank test in different risk group according to DeepSurv model. **(A)** The DeepSurv risk stratification of patients in the training cohort. **(B)** The DeepSurv risk stratification of patients in the validation cohort.
