# Supplementary figures and images for: Development and validation of machine learning models for predicting prognosis and guiding individualized postoperative chemotherapy: A real-world study of distal cholangiocarcinoma
Source: Front Oncol. 2023 Mar 15;13:1106029. doi: 10.3389/fonc.2023.1106029 (PMC10050553; doi:10.3389/fonc.2023.1106029)

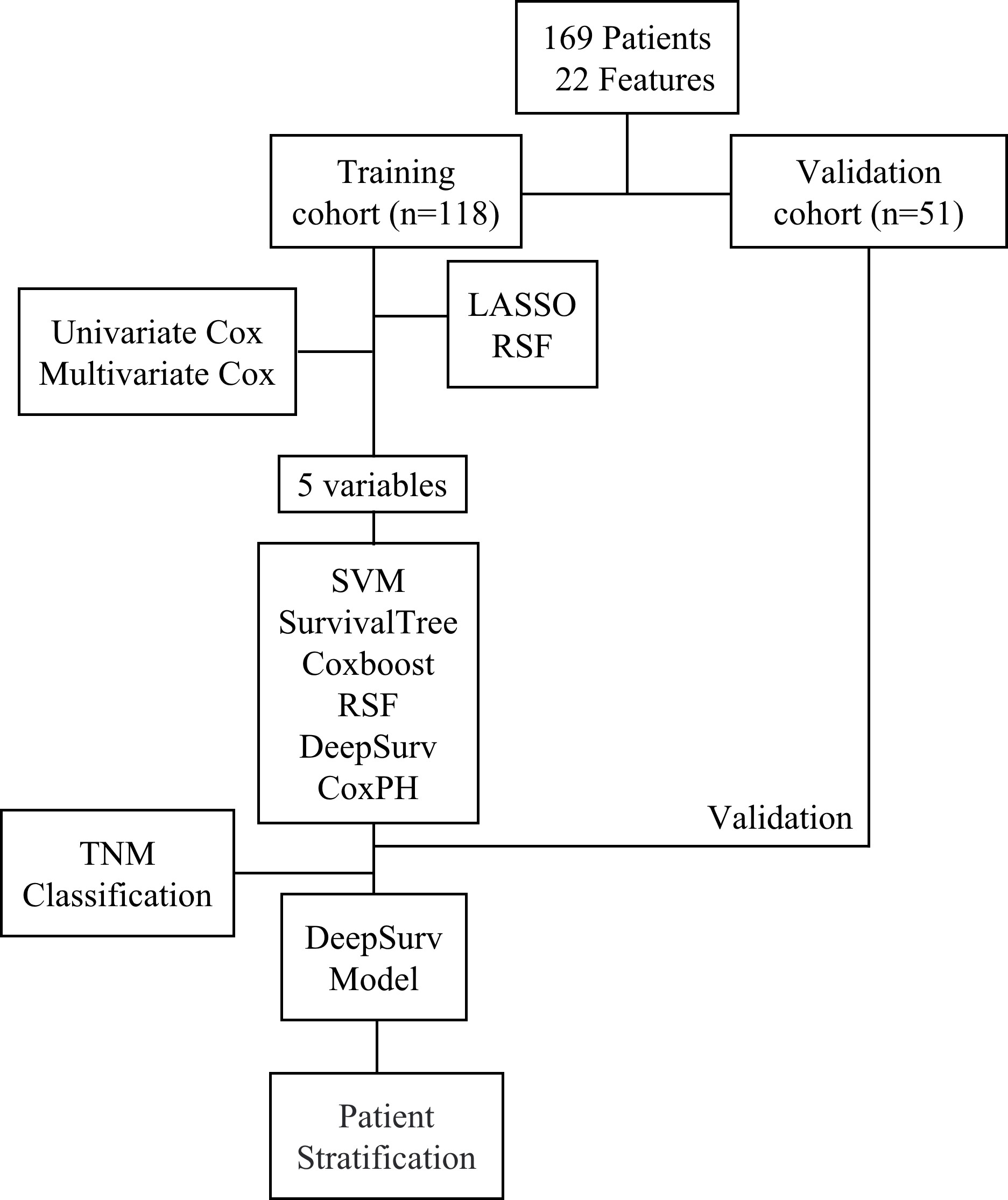

Supplement: Supplementary Figure 1 — Flow chart of this study. [file Image_1.jpeg]

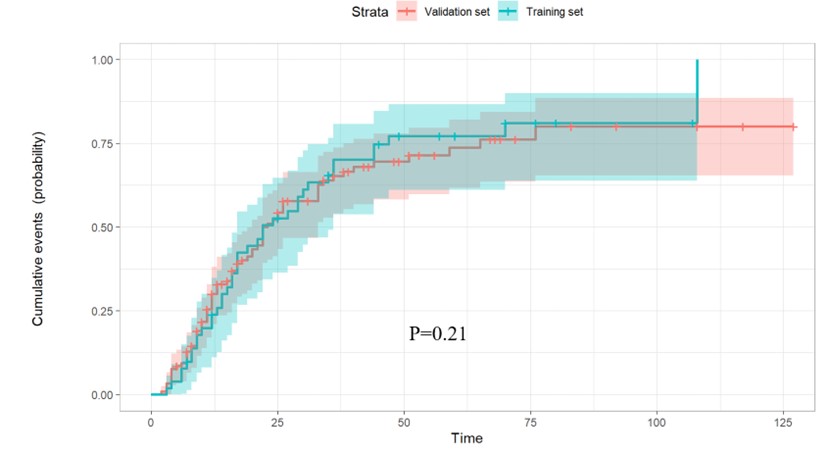

Supplement: Supplementary Figure 2 — Cumulative incidence curves of the training cohort and the validation cohort. Cumulative incidence curve in the training cohort and the validation cohort. There was no statistically significant difference between the survival of the two cohorts using the log-rank test (p = 0.21). [file Image_2.jpeg]

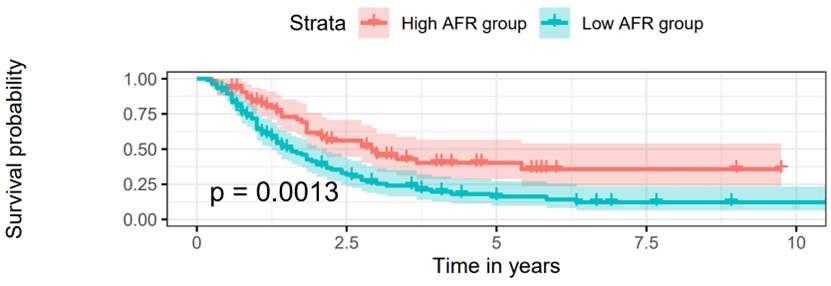

Supplement: Supplementary Figure 3 — The Kaplan–Meier analysis and log-rank test in different AFR groups. The Kaplan–Meier analysis in different AFR groups. There was a statistically significant difference between the survival of the two groups using the log-rank test (p = 0.0013). [file Image_3.jpeg]

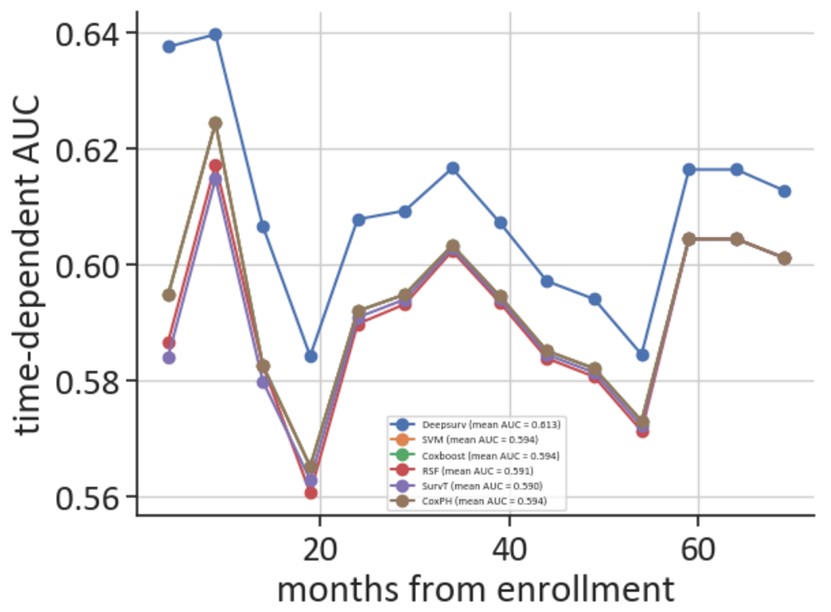

Supplement: Supplementary Figure 4 — The time-dependent ROC analysis in TNM Classification. The time-dependent ROC analysis in TNM Classification and the DeepSurv algorithm had the highest mean AUC. [file Image_4.jpeg]

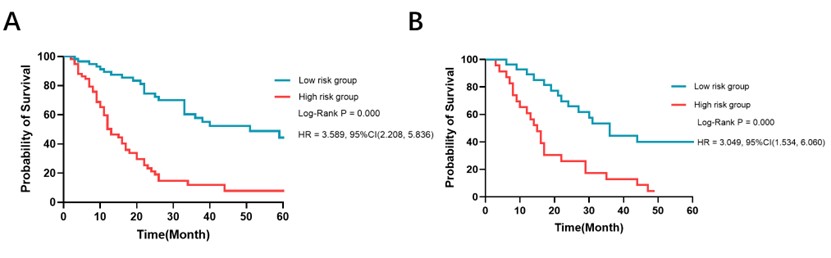

Supplement: Supplementary Figure 5 — The Kaplan–Meier analysis and log-rank test in different risk group according to DeepSurv model. (A) The DeepSurv risk stratification of patients in the training cohort. (B) The DeepSurv risk stratification of patients in the validation cohort. [file Image_5.jpeg]
