# Supplementary material for: Development and validation of machine learning models for predicting prognosis and guiding individualized postoperative chemotherapy: A real-world study of distal cholangiocarcinoma
Source: Front Oncol. 2023 Mar 15;13:1106029. doi: 10.3389/fonc.2023.1106029 (PMC10050553; doi:10.3389/fonc.2023.1106029)
Supplement: Supplementary file 6 [file DataSheet_1.pdf]

Supplementary Table 1. Hyperparameter spaces and optimal values

**Parameter**	**Search space**	**Selected value**
Learning rate	[0.01, 0.1]	0.1
Number of hidden layers	[0, 3]	2
Number of nodes	8, 16, 32, 64	32
Dropout	[0, 0.5]	0.2
Epochs	[10, 100, 200]	100
Batch size	64, 128, 256	256

Supplementary Table 2. The performance between DeepSurv model and TNM Classification.

**Model**	**C-index**		**Mean AUC**		**IBS**	
	Training cohort	Validation cohort	Training cohort	Validation cohort	Training cohort	Validation cohort
TNM	0.589	0.568	0.613	0.599	0.186	0.172
DeepSurv	0.746	0.711	0.823	0.753	0.132	0.147
